# Commentary: Greek Yogurt and 12 Weeks of Exercise Training on Strength, Muscle Thickness and Body Composition in Lean, Untrained, University-Aged Males

**DOI:** 10.3389/fnut.2019.00137

**Published:** 2019-09-10

**Authors:** Johan Jakobsson

**Affiliations:** Sports Medicine Unit, Community Medicine and Rehabilitation, Umeå University, Umeå, Sweden

**Keywords:** greek yogurt, resistance training, strength training, exercise, protein intake

## Introduction

With great interest, I read the article by Bridge et al. (1). Following a discussion on resistance training and dairy protein, the authors studied the effect of Greek Yogurt (GY) consumption in combination with 12 weeks of resistance training on strength, muscle thickness and body composition in lean, untrained, university-aged males. This commentary intends to constructively comment on the recently published article by Bridge et al. by discussing some of the results, conclusions, and the study design.

The authors are discussing the potential of GY as a viable post-exercise food, due to its high protein content. The protein in GY is mostly casein, which is discussed as a protein with unique characteristics because it can prolong plasma amino acids and enhance whole-body protein turnover due to its slow absorption (2) and thus, allowing a net positive protein balance over a prolonged period of time (3). Given the effectiveness of milk in the context of stimulating muscle protein synthesis (MPS) (4, 5) and health-related benefits of yogurt consumption (6–9) it is discussed that further studies on GY are warranted.

## Discussion

One aim of the paper was to assess if GY could be used as a whole food source of protein to support training adaptations vs. an iso-energetic control. The authors accurately acknowledge this question but become quite speculative on the beneficial effects of GY. In the introduction, the authors raised an interesting question: Would other dairy products elicit the same positive adaptations to resistance training as milk? However, this study compared GY to a carbohydrate-based pudding with no protein. One group consumed 200 g of GY (110 kcal, 20 g protein, 8 g carbohydrates) 3 times/day on training days, and 150 g of GY 2 times/day on non-training days. The control group consumed an iso-energetic, semi-solid carbohydrate-based chocolate-flavored placebo pudding (110 kcal, 0 g protein, 28 g carbohydrates).

Because the GY group received an additional 60 g of protein on training days, and 40 g on non-training days, their protein consumption (1.74 g/kg/d) was significantly higher than the control group's (1.22 g/kg/d). It is evident that an increase in protein consumption of this magnitude will increase the adaptations and muscle hypertrophy by resistance training, as shown by several studies (10–14) and extensive meta-analyses (15–17). Participants in the GY group also consumed GY before bed, which most likely resulted in a decrease in muscle protein breakdown overnight (10).

Thus, the results are expected and leaves us with the question of whether other dairy products could elicit the same positive adaptations to resistance training as milk. While the study had several strengths, such as minimal inter-tester variation, and blinded trainers, as a result of the study design, it can only be concluded that a higher protein consumption, induced by 40–60 g of protein derived from GY per day, will increase the strength and muscle thickness in untrained males in combination with resistance training during 12 weeks. Thus, high-protein bacteria fermented milk, in addition to whole-milk and milk protein concentrates such as whey, is able to potentiate adaptations from resistance training.

The study contributes to the literature of GY and protein supplementation and GY could be considered as a viable post-exercise food. However, while not the primary aim of the study, it would have been of great value to include a group consuming just milk, or whey protein, to answer the question regarding the effectiveness of other dairies in addition to milk. With equivalent protein intake across groups, a comparison of different protein sources is enabled. Thus, reducing the effects of a discrepancy in daily protein intake which will alter the adaptation to resistance training (16). The well-read authors briefly mention these matters and compare the present study with similar chronic training studies. One could interpret the findings and improved adaptations as similar to what have been seen in prior studies with milk or whey supplementation, but this is only speculative. Thus, the limitations of the study remain, and the authors make some unsubstantiated claims on the beneficial effects of GY, such as GY could be more beneficial at promoting a positive protein balance than milk.

Nevertheless, the authors state an interesting question. Therefore, further studies analyzing consumption of GY, including measurements of plasma amino acid concentrations and measurements of muscle protein synthesis, briefly mentioned by the authors, are of interest to further shed light in the context of GY consumption in combination with resistance training. By using stable isotope methods, researchers could decipher the anabolic potency of GY on the stimulation of MPS enabling more in-depth comparison of GY vs. other protein sources, and the potential of a whole-foods matrix on positively influencing the utilization of amino acids by muscle tissue (18).

## Author Contributions

JJ conceived, wrote, and revised the manuscript.

### Conflict of Interest Statement

The author declares that the research was conducted in the absence of any commercial or financial relationships that could be construed as a potential conflict of interest.
